# Association between Lipoprotein Levels and Humoral Reactivity to *Mycobacterium avium* subsp. *paratuberculosis* in Multiple Sclerosis, Type 1 Diabetes Mellitus and Rheumatoid Arthritis

**DOI:** 10.3390/microorganisms7100423

**Published:** 2019-10-08

**Authors:** Marco Bo, Giannina Arru, Magdalena Niegowska, Gian Luca Erre, Piera Angela Manchia, Leonardo A. Sechi

**Affiliations:** 1 Department of Biomedical Sciences, Section of Microbiology and Virology, University of Sassari, Viale San Pietro 43b, 07100 Sassari, Italy; 3003953@studenti.uniss.it (M.B.); magda@uniss.it (M.N.); 2 Department of Clinical, Surgical and Experimental Medicine, Neurological Clinic, University of Sassari, Viale San Pietro 8, 07100 Sassari, Italy; parentina@yahoo.it; 3 Department of Clinical and Experimental Medicine, Azienda Ospedaliero-Universitaria di Sassari, UOC di Reumatologia, Viale San Pietro 8, 07100 Sassari, Italy; e.gianluca@libero.it; 4 Centro Trasfusionale, AOU Sassari, 07100 Sassari, Italy; Pierangela.manchia@aousassari.it

**Keywords:** *Mycobacterium avium* subsp. *paratuberculosis*, cholesterol, multiple sclerosis, type 1 diabetes, rheumatoid arthritis, autoimmunity

## Abstract

Environmental factors such as bacterial infections may play an important role in the development of autoimmune diseases. *Mycobacterium avium* subsp. *paratuberculosis* (MAP) is an obligate pathogen of ruminants able to use the host’s cholesterol for survival into macrophages and has been associated with multiple sclerosis (MS), type 1 diabetes (T1DM) and rheumatoid arthritis (RA) through a molecular mimicry mechanism. Here, we aimed at investigating the correlation between humoral reactivity against MAP and serum lipoprotein levels in subjects at T1DM risk (rT1DM) grouped by geographical background and in patients affected by MS or RA. Our results showed significant differences in HDL, LDL/VLDL and Total Cholesterol (TC) levels between patients and healthy controls (*p* < 0.0001). Patients positive to anti-MAP Abs (MAP+) had lower HDL levels in comparison with Abs negative (MAP-) subjects, while opposite trends were found for LDL/VLDL concentrations (*p* < 0.05). TC levels varied between MAP+ and MAP- patients in all three assessed diseases. These findings suggest the implication of anti-MAP Abs in fluctuations of lipoprotein levels highlighting a possible link with cardiovascular disease. Further studies will be needed to confirm these results in larger groups.

## 1. Introduction

*Mycobacterium avium* subsp. *paratuberculosis* (MAP) is an intracellular pathogen with tropism for macrophages and a causative agent of severe enteritis in ruminant animals that results from its ability to elude host’s immune defense through largely unknown mechanisms [1]. The zoonotic potential of MAP has been suggested over the years by associating the bacterium with Crohn’s disease (CD) [2]. In addition, numerous other studies hypothesized its contribution to several autoimmune and neurodegenerative disorders such as type 1 diabetes mellitus (T1DM), Parkinson’s disease, Rheumatoid Arthritis (RA) and Multiple Sclerosis (MS) [3,4,5,6,7]. A recent study in sheep and cattle showed that MAP uses cholesterol as a primary carbon-based energy source during early stages of infection [8]. The uptake and trafficking of MAP in human cells seems to be favored in cholesterol-rich compartments that are slow to acidify [9]. It has also been demonstrated that MAP, similar to other pathogenic mycobacteria [10,11,12,13,14], is able to manipulate host lipid metabolism and accumulate cholesterol within macrophages to establish infection [15].

Besides structural functions in mammalian cells, cholesterol mediates numerous processes including cell signaling and pathways underlying pathogen clearance such as lysosome acidification and antigen processing [16,17]. Cellular homeostasis of lipoproteins is ensured by mechanisms soundly regulating their metabolism through biosynthesis and influx/efflux at the transcriptional and post-transcriptional levels [18,19], disturbance of which is linked to numerous pathologies characterized by chronic inflammation and cardiovascular risk [20,21,22]. In this context, either excessive amounts of intracellular cholesterol or its insufficiency may promote the intensification of inflammatory responses or adverse clinical outcomes, such as remyelination failure in the adult brain associated with hypocholesterolemia [23]. Notably, enhanced inflammatory responses due to reprogramming of cholesterol metabolism in activated cells of the adaptive immune system may lead to autoimmunity [24]. Indeed, altered levels of lipoproteins have been described in autoimmune diseases including MS, T1DM and RA, which are often complicated by atherosclerosis and cardiovascular disease (CVD) [25,26,27]. Additionally, the active phase of CD was linked to decreased cholesterol levels [28]; however, the impact of MAP presence on lipoprotein content in this disease has never been explored. Few existing studies on changes in serum cholesterol in response to MAP infection assessed this aspect of host-pathogen interaction in animals showing variable strain-specific lipoprotein levels [8,9,15,29,30]. As observed during early infection in MAP-exposed cattle [30], downregulation of low density lipoprotein (LDL) receptor following inflammation due to bacterial lipopolysaccharides was associated with increased host serum cholesterol levels [31]. Similar studies related to mycobacterial infections in humans describe serum cholesterol profiles in pulmonary tuberculosis [32], highlighting a risk of developing insulin resistance in newly-diagnosed patients [33,34].

Previously, we have demonstrated the significantly increased prevalence of antibodies (Abs) targeting MAP components and/or their human homologs in MS, T1DM and RA [35,36,37]. The aim of this study was to assess whether the presence of anti-MAP Abs in humans correlates with an imbalance in lipoprotein levels similarly to trends occurring in MAP-infected animals. For this purpose, we quantified high density lipoprotein (HDL), low density lipoprotein/very low density lipoprotein (LDL/VLDL) and total cholesterol (TC) levels in subjects at risk of T1DM (rT1DM), MS and RA selected according to their MAP-specific Abs status. Our findings show a statistical difference in lipoprotein levels between MAP-positive (MAP+) and MAP-negative (MAP-) patients providing an additional clue in favor of the theory seeing MAP involved in human pathologies. To our knowledge, this is the first study investigating a possible association between MAP-related antigens and serum cholesterol in human diseases.

## 2. Materials and Methods

### 2.1. Subjects

In the present study, the following groups were formed based on the pathological condition: 22 MS patients (1:1.4 male/female ratio; 40 years median age), 22 rT1DM patients (1:1.6 male/female ratio; 4 years median age), 22 RA subjects (1:2.7 male/female ratio; 49 years median age) and 22 healthy controls (HCs; 2.7:1 male/female ratio; 37 years median age).

MS patients diagnosed according to the revised McDonald diagnostic criteria [38] were enrolled at the Neurological Clinic of the University Hospital of Cagliari, Italy. At the time of the study, 19 patients were diagnosed as relapsing remitting MS (RRMS) and three as secondary progressive MS (SPMS). The Expanded Disability Status Scale (EDSS) values ranged from 0 to 7.0 with the average of 1.93. Demographic, clinical and laboratory features of MS are summarized in Table 1.

rT1DM subjects were recruited in Sardinia at the Department of Diabetes, St. Michele Hospital of Cagliari and in mainland Italy at the Tor Vergata University Hospital of Rome. T1DM risk was intended as disease familiarity between first-degree relatives, detection of high-risk HLA alleles and/or presence of diagnostic autoantibodies (ZnT8, GADA, IA2A, IAA and/or ICA). All subjects were free from therapy.

RA patients diagnosed according to the 2010 ACR/EULAR classification criteria [39] were enrolled at the outpatient clinic of the Rheumatology Unit, Department of Clinical and Experimental Medicine, University Hospital of Sassari, Italy. Collected data included: duration of RA, therapy (steroids, Tocilizumab, DMARDs and/or anti-TNF-α), levels of C-reactive protein (CRP), erythrocyte sedimentation rate (ESR) levels, positivity to rheumatoid factor and anti-cyclic citrullinated peptide (anti-CCP), Disease Activity Score-28 (DAS-28) and grade of disability defined through the health assessment questionnaire. Demographic, clinical and laboratory features of RA are summarized in Table 2.

HCs were recruited at the Blood Transfusion Center of Sassari, Italy.

### 2.2. Ethics Statement

The study was approved by the Ethics Committee of the Azienda Ospedaliero-Universitaria of Cagliari, Italy (prot. Num. PG/2018/5463). All participants or their legal guardians have given written informed consent. All methods were carried out in accordance with the approved guidelines.

### 2.3. Quantification of Lipoproteins in Serum Samples

3 mL of peripheral blood were drawn in serum Vacutainer tubes from MS, T1DM and RA subjects. Blood was centrifuged at 1500× *rpm* to separate serum for further quantifications of HDL (high-density lipoprotein), LDL (low-density lipoproteins), VLDL (very-low density lipoproteins) and total cholesterol (TC). Serum was aliquoted and conserved at −20 °C for short-term storage (<5 months) and at −80 °C for long-term storage (>5 months). The quantification was performed using HDL and LDL/VLDL Quantification kit (Sigma-Aldrich).

### 2.4. Enzyme-Linked Immunosorbent Assay (ELISA)

For each disease, 11 patients positive to MAP-derived antigens (MAP^+^) and 11 MAP-seronegative subjects (MAP^-^) were selected. The prevalence of MAP-specific antibodies was assessed against MAP_4027_18–32_ peptide highly recognized in MS and RA, and against at least one of the following MAP peptides homologous to zinc transporter 8 (ZnT8) or proinsulin fragments: MAP3865c_133–141_, MAP3865c_125–133_, MAP2404c_70–85_ and MAP1,4αgbp_157–173_.

Indirect ELISA to detect antibodies (Abs) against MAP peptides was performed as described previously [7]. The optical density (OD) was read at a wavelength of 405 nm using SpectraMax Plus 384 microplate reader (Molecular Devices, Sunnyvale, CA 94089, USA). The cut-off value for positivity in each assay was calculated based on ROC analysis with specificity set at > 90% and sensitivity chosen accordingly.

### 2.5. Statistical Analysis

All data were analyzed through GraphPad Prism 6.0 software (San Diego, CA, USA). Differences between quantitative variables and Abs levels were analyzed using the Mann–Whitney and the Kruskal–Wallis tests to compare two and more groups, respectively. Differences with *p* < 0.05 were considered statistically significant.

## 3. Results

**Figure 1:**
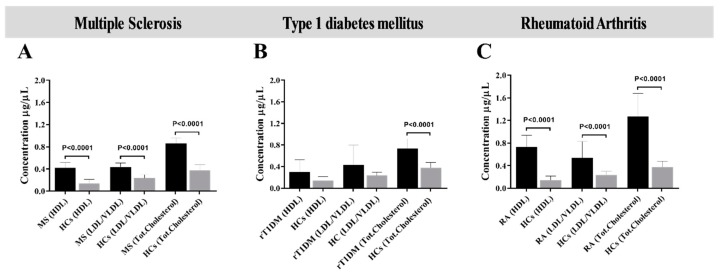
Levels of serum lipoproteins determined through disease-specific analysis. Bars show values were determined in samples from patients (**A**) with MS, (**B**) at risk of T1DM (rT1DM) and (**C**) affected by RA, each analyzed with reference to concentrations obtained for healthy controls (HCs). p-values are specified for each group when statistically significant. Standard deviation is shown for each bar.

Upon the analysis of differences in lipoproteins levels in each disease group, MS and RA patients showed significantly increased levels of HDL, LDL/VLDL and TC in comparison with HCs (*p* < 0.0001, Figure 1A,C). These results are in line with studies in sheep challenged with MAP where total serum cholesterol levels were elevated at 9 weeks post-inoculation (wpi) respect to uninfected animals [8]. In contrast, statistically significant difference between rT1DM and HC subjects was found only for total cholesterol (*p* < 0.0001, Figure 1B).

Next, we compared the concentrations of HDL, LDL/VLDL and TC in the sera of MAP+ and MAP- subjects of each pathological condition. The following associations were sought to understand whether anti-MAP Abs are indicative of a possible past exposure to this *Mycobacterium* sp. or an ongoing silent infection associate with quantity variation of the host lipids: MS MAP+ versus MS MAP- subjects; MS MAP+ versus HCs MAP+ subjects; MS MAP- versus HCs MAP- subjects. The same association analysis was done for rT1DM and RA subjects.

**Figure 2:**
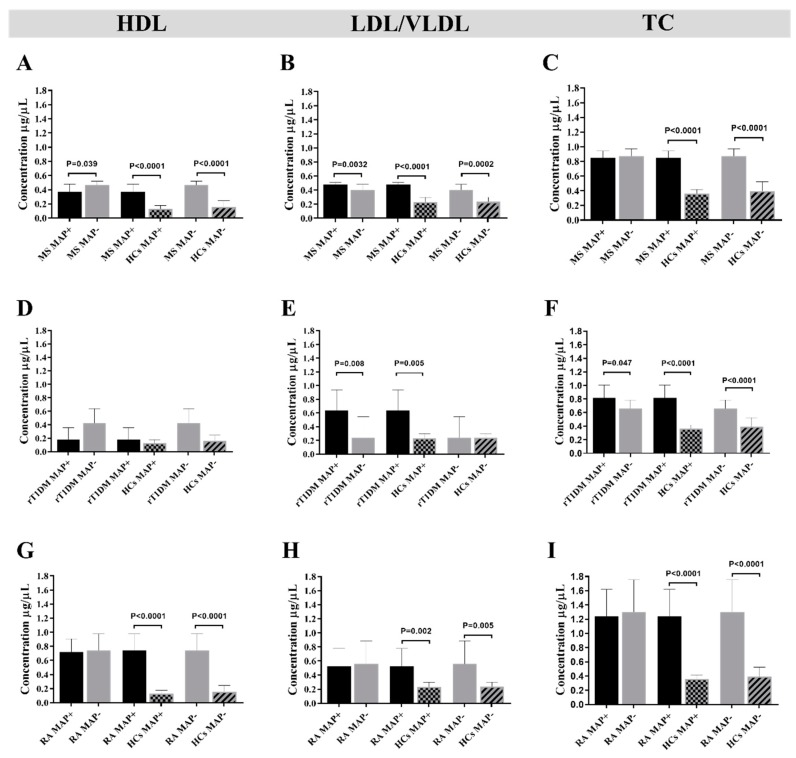
Disease-related concentrations of high density lipoprotein (HDL), low density lipoprotein (LDL) and total cholesterol (TC) assessed based on positivity to MAP antigens. The analysis was performed by comparing MAP+ versus MAP- subjects among patients affected by MS (**A**–**C**), at risk of T1DM (rT1DM) (**D**–**F**) and RA subjects (**G**–**I**). Additionally, a comparison of patients and healthy controls (HCs) was carried out for the same MAP-related serological status. Statistical significance is reported above relative bars when attained. For each group, standard deviation is indicated.

In MS, a significant difference in HDL levels was found between MAP+ and MAP- patients (*p* = 0.0398, Figure 2A). MS MAP+ showed higher HDL concentrations when compared with HCs MAP+, (*p* = 0.0001, Figure 2A) and similar trends were observed between MS MAP- and HCs MAP- (*p* < 0.0001, Figure 2A). It is interesting to note that the levels of HDL, LDL and TC were lower in HCs MAP+ than HCs MAP-, although statistical significance was not attained (Figure 2A–C). The respective analysis performed for LDL/VLDL provided significant results between all groups analyzed (Figure 2B). Regarding TC concentrations, no difference was found between MS MAP+ and MS MAP- but significantly higher levels were registered for MS MAP+ comparing to HCs MAP+ (*p* < 0.0001, Figure 2C) and for MS MAP- versus HCs MAP- (*p* < 0.0001, Figure 2C).

In summary, MS MAP+ subjects are characterized by a significant decrease in HDL levels, an increase in LDL/VLDL (Figure 2A,B) and no difference in TC levels. It is to be highlighted that the concentrations of HDL, LDL/VLDL and TC in MS patients were markedly elevated respect to HCs. Evidence has showed that HDL, LDL and TC variations were associated with MS progression [25,40,41]. For this reason, we performed an association analysis of serum HDL, LDL and TC levels and disability status in MS MAP+ and MAP- patients. Even though the general association analysis between EDSS (Expanded Disability Status Scale) and TC levels showed no correlation (R^2^ = 0.06), it is noteworthy that after subdivision into MAP+ and MAP- groups, we obtained a higher correlation coefficient between EDSS and TC levels in MS MAP+ than MS MAP- (R^2^ = 0.14 versus R^2^ = 0.03). Overall, the highest correlation was observed between EDSS and LDL in MAP+ (R^2^ = 0.55). EDSS correlated well with HDL in MS MAP+ (R^2^ = 0.34), while low coefficient was obtained for the same variables in MS MAP- (R^2^ = 0.05). In addition, we performed the correlation analyses between the Abs response to MAP_4027_18–32_ peptide and TC, HDL and LDL levels, which resulted in higher coefficients for TC and HDL levels in MS MAP+ (R^2^ = 0.358 and R^2^ = 0.493, respectively). No correlation with TC, HDL and LDL levels was found in MS MAP- subjects. Similarly, the assessment of possible lipoprotein variations in patients receiving cortisone therapy revealed no difference.

In rT1DM subjects, no difference in HDL levels was observed between the analyzed groups (Figure 2D). In contrast, we found significantly lower LDL/VLDL concentrations among rT1DM MAP- compared with rT1DM MAP+ subjects (*p* = 0.0080, Figure 2E). Statistically significant reduction of TC levels among MAP- subjects were obtained in all association groups: rT1DM MAP+ versus rT1DM MAP-, rT1DM MAP+ versus HCs MAP-, rT1DM MAP- versus HCs MAP-, with the respective *p*-values: *p* = 0.0471, *p* < 0.0001 and *p* < 0.0001 (Figure 2F).

**Figure 3:**
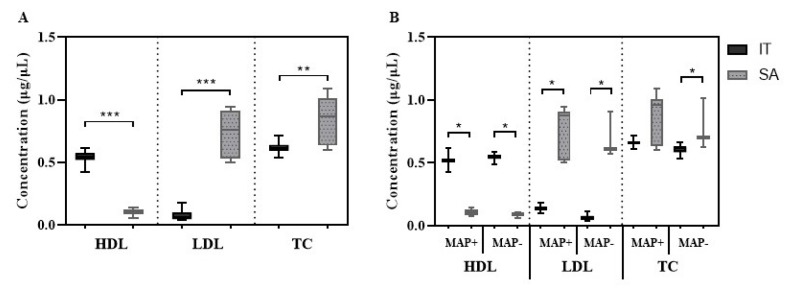
Concentrations of lipoproteins in subjects at risk of T1DM (rT1DM) grouped according to their geographic provenience. (**A**) Comparison between patients enrolled in mainland Italy (IT; *n* = 10) and Sardinia (SA; *n* = 12). (**B**) Differences in lipoprotein levels between IT and SA participants grouped according to their serological response to MAP antigens. Mean and standard deviation are indicated for each bar. Upon the analysis of lipoprotein levels in RA, our results showed a statistical difference in HDL concentrations between RA MAP+ and RA MAP- and when comparing RA MAP- with HCs MAP- (*p* < 0.0001, Figure 2G). Insignificantly lower HDL concentrations were found among RA MAP+ respect to RA MAP- (Figure 2G). Increased levels of LDL/VLDL were observed in RA MAP+ versus HCs MAP+ (*p* = 0.0024, Figure 2H) and in RA MAP- versus HCs MAP- (*p* = 0.0052, Figure 2G); however, a comparison between RA MAP+ and RA MAP- showed no difference. Similar results were obtained for TC (Figure 2I), with strikingly lower levels among HCs regardless of anti-MAP Abs status (*p* < 0.0001, Figure 2I).

After classifying patients based on their geographic provenience, we observed significantly higher levels of HDL (*p* < 0.0001) and lower LDL (*p* < 0.0001) and TC (*p* = 0.0044) concentrations in the group from mainland Italy compared to samples collected in Sardinia (Figure 3A), which were mirrored by trends in MAP+ and MAP- subjects considering location of enrollment (Figure 3B).

## 4. Discussion

MS, T1DM and RA are among complex autoimmune diseases developing through a multifaceted interplay between genetic determinants, environmental factors and the immune system. Viral and bacterial infections with host’s molecular components play a crucial role in triggering autoimmunity in directed manner or by activating secondary immune responses exacerbating the ongoing autoimmune process. Our previous studies support the hypothesis that MAP may be a contributing agent in the etiology of MS, T1DM and RA through cross-reactivity and molecular mimicry phenomena [5,35,36,37,42]. Here, we investigated whether humoral responses against MAP correlate with altered lipoprotein profiles typical to these diseases. The outcomes of this study have shown variations in serum cholesterol levels associated with the presence of anti-MAP Abs.

Genome-wide association study (GWAS) identified over 100 distinct genetic variants associated with MS predisposition [43] that show a genetic modulation of lipid profiles in disability progression. In addition, recent discoveries showed that other genetic contributors to MS development reside within the ubiquitin-proteasome system that represents an immensely important pathway in protein degradation [44]. Less is known about environmental MS contributors and their link with genetically conferred susceptibility to infections. Recent evidences associate serum lipid levels and lipid-related polymorphisms with disability progression in MS patients [38]. Cholesterol synthesis appears crucial during remyelination of neuroglia [45], while its excess in plasma may aggravate neuronal cell damage [46], thus conflicting results have been obtained for lipoprotein concentrations and degree of disability in this disease [25,41,47]. Our results indicated higher levels of serum lipoprotein levels in MS patients, showing lower HDL concentrations among MAP+ subjects which correlated with higher EDSS. It is possible that lipid-related SNPs inducie imbalance of cholesterol, thereby favoring mycobacterial survival in macrophages. This hypothesis would need a further screening for relevant gene polymorphisms and assessment to which extent MAP may alter lipoprotein homeostasis. A study in C57BL/6J mice highlighted the ability of a myelin oligodendrocyte glycoprotein MOG_35–55_ peptide from heat-killed MAP to induce experimental autoimmune encephalomyelitis (EAE), which is considered a model condition for MS studies [48]. In addition, EAE was more severe in MAP-immunized mice than in animals treated with Freund’s Complete Adjuvant (CFA)—a nonspecific stimulator of the immune response. Still, MAP components were able to activate a strong proliferative T cell response.

Differences in lipoprotein levels between rT1DM patients with distinct biogeographical background indicating possible genetic and environmental determinants are noteworthy. Low HDL and raised LDL levels are typical features of young rT1DM patients at high CVD risk and were detected among Sardinian participants, while opposite trends were displayed by subjects from mainland Italy. The island of Sardinia is characterized by the second highest prevalence of T1DM worldwide [49] and the by peculiar genetic heritage of local populations due to long lasting genetic isolation [50]. Over 60% of Sardinian livestock herds seem to be infected with MAP [51]; however, these estimates may reach more elevated numbers given the lack of official registers and monitoring strategies. Markedly high concentrations of LDL in Sardinian rT1DM patients compared to the study group enrolled in Rome reflect observations in MAP-infected animals and may be indicative of past exposure to MAP in combination with gene variants facilitating or enhancing the effects of infection [8]. On the other hand, slightly differing concentrations of serum lipoproteins between MAP+ and MAP- subjects within the same geographically-related group may present a temporary picture of a latent infection when MAP is not actively modulating lipoprotein profiles and should be further investigated in larger cohorts. Such observations in the phase when clinical symptoms of diabetes are still to be developed indicate that the effects of MAP infection on lipoprotein profiles may have initiated long before disease onset with a possible implication in T1DM pathogenesis and/or severity.

Genetic regulation of lipid metabolism, particularly in the context of gene–environment interaction, has not been examined in the RA population. This may be particularly important, since lipid alterations appear to predate the diagnosis of RA [27] and may be exacerbated during initial phases of MAP infection. Results obtained for RA cohort in this study are discordant compared to literature, although similar differences in lipoprotein levels have been described for distinct animal species. Upon exposure to different MAP strains, a significant increase in total serum cholesterol has been observed after 9 weeks post infection (wpi) in sheep, while cattle showed an opposite trend at 13 wpi [8]. The way in which cholesterol is exploited by MAP in varying time lags depends, therefore, on the host and host-specific mycobacterial strains. Lower but not significant lipoprotein levels detected among RA MAP+ patients compared to RA MAP- in our study may reflect MAP infection phase that at the initial stage is silent and difficult to diagnose [52]. A higher HDL, LDL and TC levels were found in RA patients in comparison to HCs independently from MAP positivity; however, a slightly lower level of lipoproteins was observed in MAP positive in comparison to MAP negative patients.

The evaluation of such interplay may be complex, as lifelong therapy administered to RA patients targets elements of the immune system resulting in suppressed responses to antigens in general and may affect cholesterol metabolism [53].

Considering the complicated relationship between MAP and other factors involved in autoimmune processes that lead to an imbalance in lipoprotein levels, follow-up studies and employment of murine models representing the corresponding diseases (MS, T1DM and RA) will allow to monitor the relationship between anti-MAP immune responses and cholesterol levels and to explore mechanisms through which this *Mycobacterium* sp. may favor pathological phenotypes. Supposedly, dependence of MAP survival on cholesterol is not only confined to the first stage of infection, but continues later during possibly prolonged silent state which may be dominant in non-primary hosts such as humans. A slow release of cholesterol previously accumulated by MAP inside macrophage may be released during the gradual killing of the pathogen, thus leading to a variation in lipoprotein levels. A successful isolation of MAP along with strain characterization would shed light on strain-specific functional differences in lipid regulation and immune responses. This would help to position MAP in complex molecular pathways underlying described autoimmune conditions. For the time being, preliminary results presented here need to be confirmed in a larger cohort and an accurate evaluation of confounding factors such as therapy, genetic predisposition and age/lifestyle-related cholesterol levels.

## Figures and Tables

**Table 1:** Demographic and clinical characteristics of MS patients and HCs.

	MS	HCs
	*n* = 22	*n* = 22
Age, years	39.77 ± 12.53	36.72 ± 11.59
Female, n (%)	13 (59.09)	16 (72.72)
Cortisone	12	
No cortisone	10	
EDSS	2.53 ± 2	
Relapse, n (%)	13 (59.09)	
RRMS, n (%)	19 (86.36)	
SPMS, n (%)	3 (13.63)	

MS, multiple sclerosis; HCs, healthy controls; EDSS, Expanded Disability Status Scale; RRMM, Relapsing-Remitting MS; SPMS, Secondary progressive MS.

**Table 2:** Demographic and clinical characteristics of RA patients and HCs.

	RA	HCs
	*n* = 22	*n* = 22
Age, years	49.3 ± 8.5	36.72 ± 11.59
Female, n (%)	16 (72.7)	16 (72.72)
ESR, mm/h	18.9 ± 15	
CRP, mg/dL	1.01 ± 0.9	
DAS28 score	3.15 ± 1.3	
HAQ score	0.7 ± 0.6	
ACPA positivity, n (%)	12 (54.5)	
RF positivity, n (%)	13 (59)	
Steroid use, n (%)	12 (54.5)	
Steroid dose, mg/day	1.58 ± 2.6	
DMARDs use, %	14 (63.6)	
TNFi use, %	4 (3.3)	
Tocilizumab use, %	7 (58.3)	

RA, rheumatoid arthritis; HCs, healthy controls; ESR, erythrocyte sedimentation rate; CRP, C-reactive protein; DAS-28, disease activity score-28 joints; HAQ, health assessment questionnaire; ACPA, anti-citrullinated peptide antibodies; RF, rheumatoid factor; DMARDs, disease-modifying anti-rheumatic drugs; TNFi, tumor necrosis factor-alpha inhibitors.
